# 

**Published:** 1887-05

**Authors:** 


### CONTENTS.

ORIGINAL COMMUNICATIONS—

Lectures on Diseases of the Skin. By Henry Wile, M. D., Atlanta, Ga. .... 129

Remittent Fever—Its Prompt Arrest and Effective Treatment. By John T. Jones, M. D., New Orleans .... 137

CLINIC REPORTS—

Granular Conjunctivitis with and without Pannus. By W. Cheatham, M. D., Louisville .... 142

SOCIETY REPORTS—

Medical Association of Georgia .... 147

CORRESPONDENCE—

Our New York Letter .... 178

A Trip Through Florida .... 182

Separation of Symphysis Pubis during Parturition .... 184

EDITORIAL—

The Medical Association Banquet .... 186

The Medical Association of Georgia .... 190

Dr. Wile's Death .... 191

Items .... 193

